# Methodology for In Situ Microsensor Profiling of Hydrogen, pH, Oxidation–Reduction Potential, and Electric Potential throughout Three-Dimensional Porous Cathodes of (Bio)Electrochemical Systems

**DOI:** 10.1021/acs.analchem.2c03121

**Published:** 2023-01-30

**Authors:** Sanne M. de Smit, Jelle J. H. Langedijk, Lennert C. A. van Haalen, Shih Hsuan Lin, Johannes H. Bitter, David P. B. T. B. Strik

**Affiliations:** † Environmental Technology, Wageningen University and Research, Wageningen 6708WG, The Netherlands; ‡ Biobased Chemistry and Technology, Wageningen University and Research, Wageningen 6708WG, The Netherlands

## Abstract

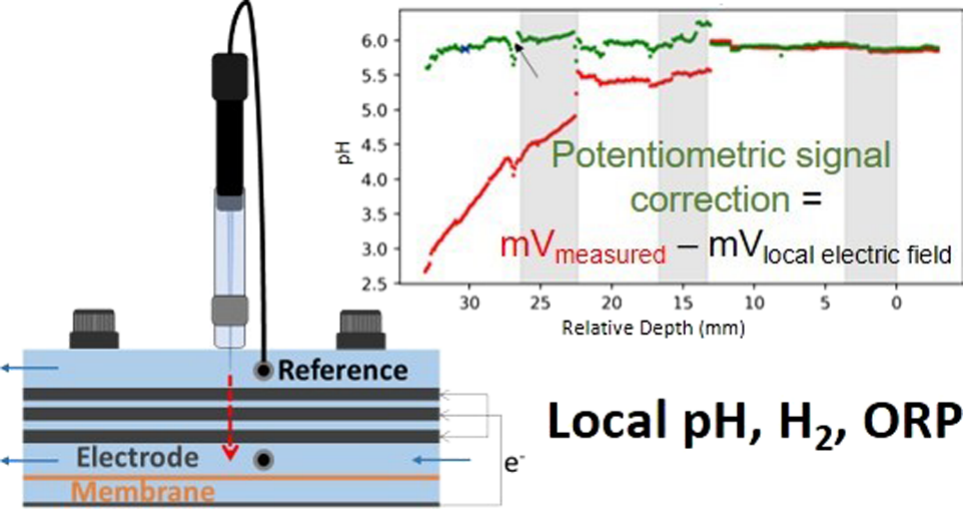

We developed a technique based on the use of microsensors to measure pH and H_2_ gradients during microbial electrosynthesis. The use of 3D electrodes in (bio)electrochemical systems likely results in the occurrence of gradients from the bulk conditions into the electrode. Since these gradients, e.g., with respect to pH and reactant/product concentrations determine the performance of the electrode, it is essential to be able to accurately measure them. Apart from these parameters, also local oxidation–reduction potential and electric field potential were determined in the electrolyte and throughout the 3D porous electrodes. Key was the realization that the presence of an electric field disturbed the measurements obtained by the potentiometric type of microsensor. To overcome the interference on the pH measure, a method was validated where the signal was corrected for the local electric field measured with the electric potential microsensor. The developed method provides a useful tool for studies about electrode design, reactor engineering, measuring gradients in electroactive biofilms, and flow dynamics in and around 3D porous electrodes of (bio)electrochemical systems.

Electrochemical technology offers a clean and powerful tool for both treatment of waste streams and chemical synthesis.^1^ (Bio)electrochemical systems catalyze (microbial) conversions by applying an electric potential to an electrode on which microbes grow. The microbes use the applied energy directly as electrons or indirectly as hydrogen, which is formed at the cathode from electrons and protons.^2,3^ Since most conversions occur at the electrode surface, local gradients of, e.g., protons and hydrogen with concentrations different from the bulk can be expected. Most often in (bio)electrochemical studies, only bulk conditions are measured, which can be non-representative for the local conditions around the electrode surface.^4^ Several theoretical studies have modeled the local conditions near the cathode,^5,6^ but practical support for these studies is rare. To measure local concentration gradients, microsensors could form powerful tools. Microsensors have a thin tip (down to 1 μm), which allows measurements with the same spatial resolution as the tip size. The sensors can be moved along a profile axis to measure gradients.^7,8^ Microsensors have been applied in many different fields, including biochemistry,^9,10^ plant science,^11−13^ microbiology,^14,15^ and biomedicine,^16,17^ but their application in electrochemical systems remains limited.

The application of microsensors in electrochemical systems is expected to be suitable by application of amperometric microsensors, which measure a current signal resulting from a redox reaction on the microelectrode surface. These Clark-type^18^ microsensors are used to measure H_2_, H_2_S, O_2_, NO, N_2_O, and CO_2_^8,19^ gradients in biofilms growing on 2D electrodes.^8,20^ To measure, e.g., pH, oxidation–reduction potential (ORP), and electric field potential, potentiometric sensors are used, which measure a potential drop over the sensor tip membrane between the sensor electrode and an external reference electrode.^8,21^ Despite their successful application in the beforementioned fields, the application in electrochemical systems is limited due to signal interference when placed in an electric field^22^ with significant distance (several mm) between the sensor electrode and external reference electrode tip.^23,24^

To use potentiometric microelectrodes for analysis of local gradients in (bio)electrochemical systems, the interference from the electric field needs to be tackled. The best way to tackle the issue would be to minimize the distance between the sensor electrode and the reference electrode.^23,24^ Some studies used so called “combined sensors” to measure pH in electrochemical biofilms. In these custom-made sensors, the reference and measuring electrode were built in the same sensor, connected with a conductive liquid.^8^ Although this gave reliable results, the thicker microsensor tip did not allow for the sensor to be moved over distances longer than 600 μm without piercing millimeter-wide holes in the biofilm.^23,25^ The short distance was enough to measure inside biofilms on 2D electrodes but could not be used to measure inside several millimeter or even centimeter-thick 3D porous electrodes typically used in bioanodes or biocathodes.^26−28^

In this study, a methodology for microsensor application in (bio)electrochemical systems was developed to measure gradients in the electrolyte and, for the first time, throughout porous 3D electrodes. The methodology development consisted of three steps. First, a reactor was designed, with key features that allow microprofile measurements over a range of several centimeters, keeping anaerobic conditions and continuous leak-free liquid electrolyte recirculation of 10 L/h. Practical tips and protocols (with video instructions) are provided for the use and careful handling of the microsensors to facilitate future use. Second, the reactor design was used to show a microprofile of H_2_ gradients in the reactor. Third, a correction method is presented and validated to overcome the interference of electric field during potentiometric microsensor measurements. With this method, potentiometric microsensors can finally be applied for accurate gradient measurements in (bio)electrochemical systems, even with high current (−10 kA/m^3^). The correction method was used to show gradient profiles of the electric field potential, ORP, and pH. The profiles in the study showed significant differences between bulk and local conditions at the electrode surface, which highlights the importance of the presented method and its possible application for mass transfer studies.

## Experimental Section

### Reactor Setup

**Figure 1:**
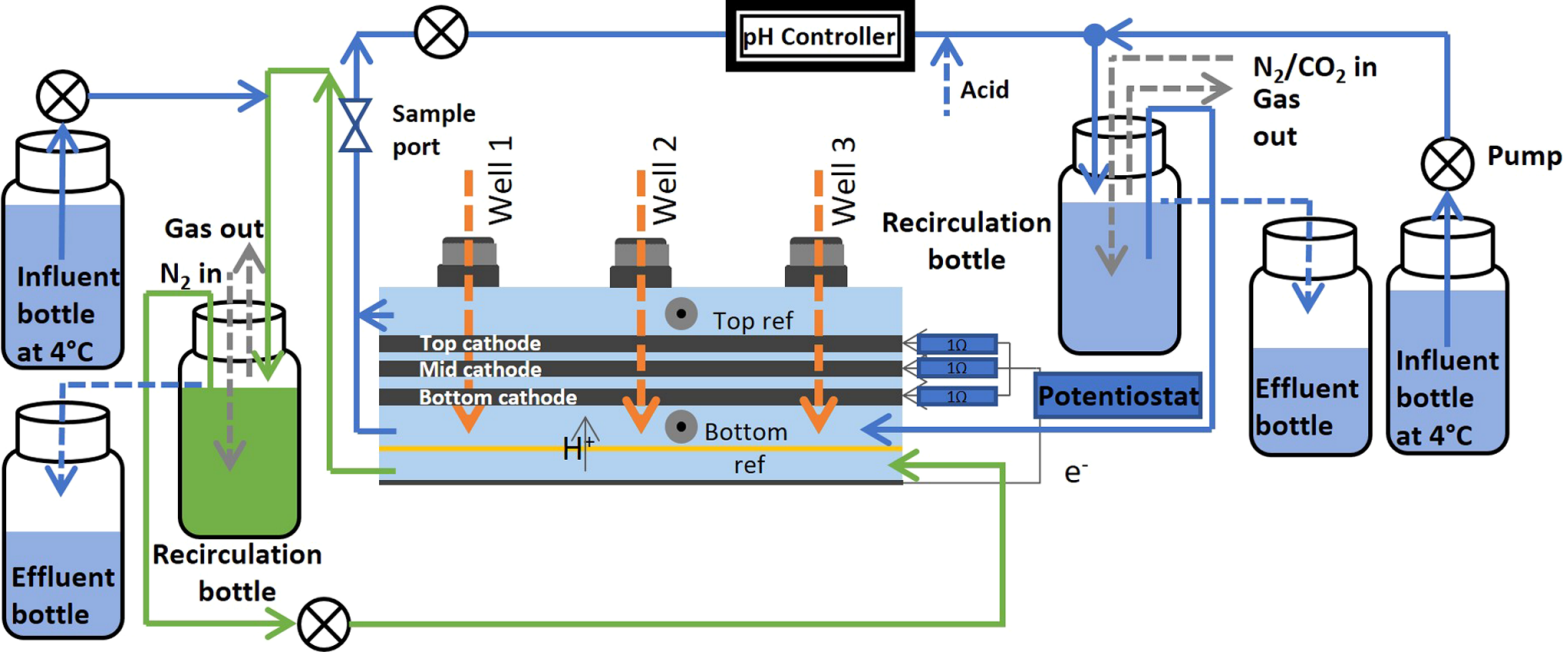
Setup of the electrochemical cell with recirculation, influent, effluent, and pH control. The anolyte recirculation is shown in green for viewing purposes.

All measurements of this study were performed in an electrochemical CO_2_-fed reactor. The electrochemical reactor consisted of an anode (Ti/Pt-Ir MMO, thickness 1 mm, Magneto Special Anodes BV, Netherlands) and three cathode layers (graphite felt, thickness 3 mm, Rayon Graphite Felt, CTG Carbon GmbH, Germany), separated by layers of three spacers to study three different distances from the anode. The anode and cathode compartments of the cell were built with Plexiglas flow-through plates with 21.3 cm^2^ psa and separated by a cation exchange membrane (Fumasep FKS, Fumatech BWT GmbH, Germany). The three cathode layers were separated with spacer layers (Figure 1, Figure S1) and connected in parallel with a titanium wire (0.8 mm-thick, grade 2, Salomon’s metalen, the Netherlands) with 1 Ω between each connection and the working electrode plug (Figure 1). The graphite felt was a non-microporous, low surface area material (<1 m^2^/g), determined by N_2_ physisorption. Since the microprofiles would be made throughout the cathode layers, the cathodes were constructed in such a way that the microsensor tips would not touch the Ti wire or spacers (Figure S1C). The construction of the cathode layers is shown in Movie S1.

Between the cathode and the membrane, a “bypass outlet” was placed at the outflow side of the electrochemical cell (left in Figure S1A). The function of the “bypass outlet” was to remove hydrogen gas that would accumulate below the cathode at current densities of −10 kA/m^3^ cathode, hindering proton transfer between the anode and cathode. The catholyte flow distribution between the two outflow ports above and underneath the cathode was 1:1, calculated from flow rate measurements at the reactor outlets. The details of the reactor operation parameters are described in the SI, section “reactor operation”.

### Microsensors and Profiling

A laboratory stand (LS18), micro-manipulator (MM33-2), motor-driven micro-manipulator stage (MMS), and motor controller (MC-232) were combined for precise manipulation of microsensors (Unisense A/S, Denmark). All equipment was installed and operated corresponding to the manuals. A H_2_ microsensor (H2-50), pH microelectrode (pH-50), oxidation–reduction potential microelectrode (RD-50), reference microelectrode (REF-100), and electric potential electrode (EP-100) were used for microprofiling, all with an indicated tip size of 40–60 μm (all from Unisense A/S, Denmark). The relative sensor lengths were determined under the macroscope to enable combination of different sensor measurements in plots. For the potentiometric microsensors, two external capillaries were installed 5 mm above (fixed ref TOP, Figure S1A) and 7 mm below the cathodes in the reactor (fixed ref BOTTOM, Figure S1A) additional to the reference used by the potentiostat. The capillaries were filled with gelified 3 M KCl and connected to Ag/AgCl reference electrodes via tubing filled with liquid 3 M KCl. The calibration and measurements corresponded to the manuals, combined with the microprofiling setup (SI section “protocol microsensor calibration” and “protocol profiling”). SensorTrace Profiling and Logging software (Unisense A/S, Denmark) were used in this study. Further details about how a profile was measured will follow in the Results section.

## Results

### Key Features of Reactor and Sensors to Allow Microprofiling

#### Reactor Design

**Figure 2:**
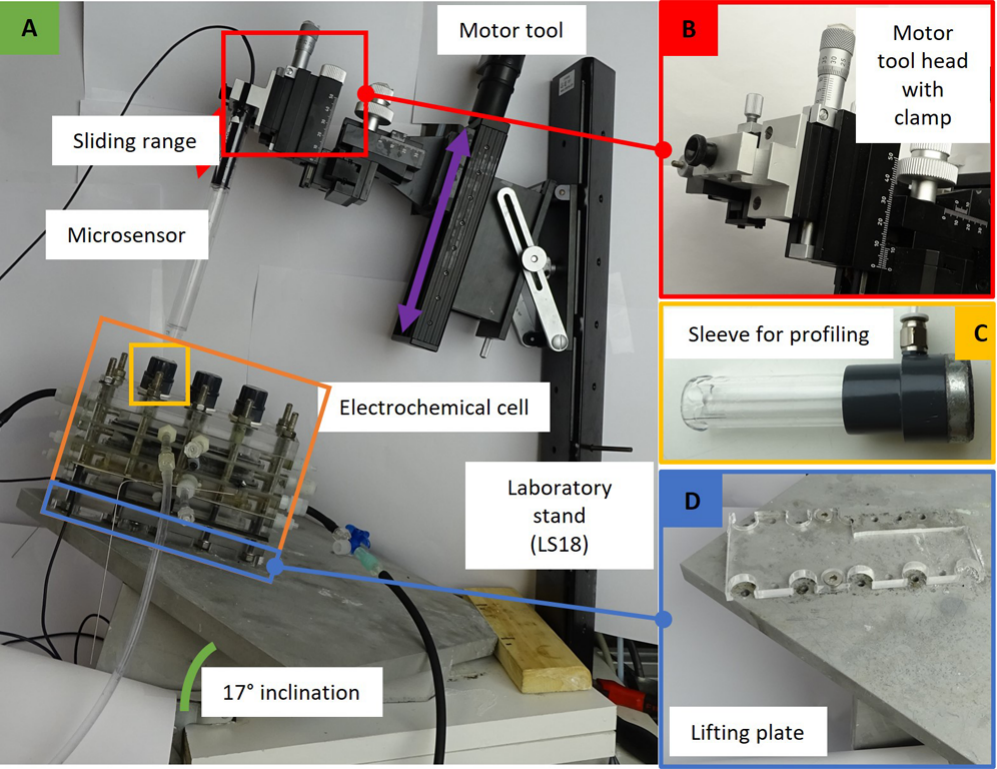
Setup for making microprofiles in a (bio)electrochemical reactor. The electrochemical reactor was placed on a lifting plate (D) connected to a ground plate with a 17° inclination (A). To make a microprofile, a microsensor is placed in the motor tool head clamp (B) and entered into the reactor via a sleeve (C).

To allow for in situ measurements with microprofiling sensors under continuous leak-free electrolyte recirculation, the setup contains some key features (Figures 1 and 2A). The electrochemical cell was fixed horizontally with a 17° angle on a ground plate to allow for gas to escape from the higher outlets and allow microprofiling with the microsensors perpendicular to the graphite felt cathode (Figure S1C, Figure 2A). The outer plate of the electrochemical cell was replaced with a Plexiglas closing plate with three wells (Figure S1A). The construction of the electrochemical reactor cell is shown in Movie S3. The inlet and outlet tubes of the catholyte compartment in the electrochemical cell were equipped with switchable three-way valves, for closing of the catholyte compartment while installing microsensors.

To measure a microprofile, the catholyte recirculation was shortly stopped to replace the well caps with a “sleeve” (Figure 2C) to enter the microsensor. The microsensor neck was greased with silicone grease to ensure a watertight seal between the neck and the sleeve. The silicone grease allowed electrolyte leak-free sensor moving. After securing the microsensor in the sleeve, the recirculation was switched on again and a profile could be made, during which the microsensor was brought down with a motor tool (default velocity and acceleration of 1000 μm/s and 1000 μm/s^2^, step size 100 μm) (Figure 2B) to pierce the graphite electrode and profile a gradient through the cathode layers until the membrane inside the cell. A full detailed protocol is described in SI section “protocol profiling” and shown in Movie S2.

### Microsensors Used for Profiling

Before measuring microprofiles, the microsensor characteristics were tested (Table 1). In this study, four different sensors were used; one amperometric sensor to measure H_2_, and three potentiometric sensors to measure electric field potential (EP), oxidation–reduction potential (ORP), and pH. The amperometric H_2_ sensor was used to test the feasibility of measuring microprofiles with the presented reactor design. The potentiometric sensors were used to develop accurate microsensor measurements in the electric field (Table 1, signal correction), which will be explained in more detail in the Correction for Electric Field Interference in Potentiometric Measurements Section.

**Table 1:** Overview of Different Microsensors Used in This Study with the Measured Value and the Used Stabilization and Measurement Times Used in This Study^a^

sensor	specs	measured	external reference	stabilization time (s) (this study)	measuring time (s) (this study)	unit	range (unit)	detection limit (unit)	signal correction (this study)
hydrogen	H2-50	local pH_2_ measured as current, converted to mV by amplifier		10	5	μmol/L	0–800	0.3	no
electric field potential	EP-100	potential difference between Ag/AgCl electrode and reference electrode	Ag/AgCl	10	5	mV	n.a.	n.a.	no
oxidation–reduction potential	RD-50	oxidation–reduction potential: solution tendency to be oxidized or reduced in mV	Ag/AgCl	3	1	mV	n.a.	0.1	mV_measured_ – mV_local electric field_
pH	pH-50	potential difference caused by proton concentration difference between sample and inside tip	Ag/AgCl	10	5		2–10	0.01	mV_measured_ – mV_local electric field_

a The microsensor tip sizes correspond to the number in the specs code, shown in μm. All sensors are from Unisense A/S, Denmark. The signal correction applies to measurements within electric fields.

First, the response times of different sensors were determined by moving the sensors to different heights in the system and measuring the signal over time (Figures S3–S5). Most signals were stable after 5 s, so this time was increased to 10 s stabilization time and 5 s measuring time during the measurements to avoid instability offsets by, e.g., hydrogen bubbles (Table 1).

### Accurate Cathode Position Determination with ORP Sensors

Apart from the technical differences between the sensors shown in Table 1, the sensor tips also showed visual differences (Figure S2). Most sensor tips were made of glass (H_2_, EP, pH), but the ORP sensor had a metal tip. The ORP microsensor measured the cathode potential when the tip was in contact with the graphite felt layers. The cathode potential values differed significantly from the electrolyte ORP, so the ORP profiles showed clearly the position of the cathode layers. Therefore, the cathode layer positions were determined by the ORP measurements and used in the profile plots of the other sensors.

### Local Hydrogen Concentration Gradients Measured with the Microsensor

**Figure 3:**
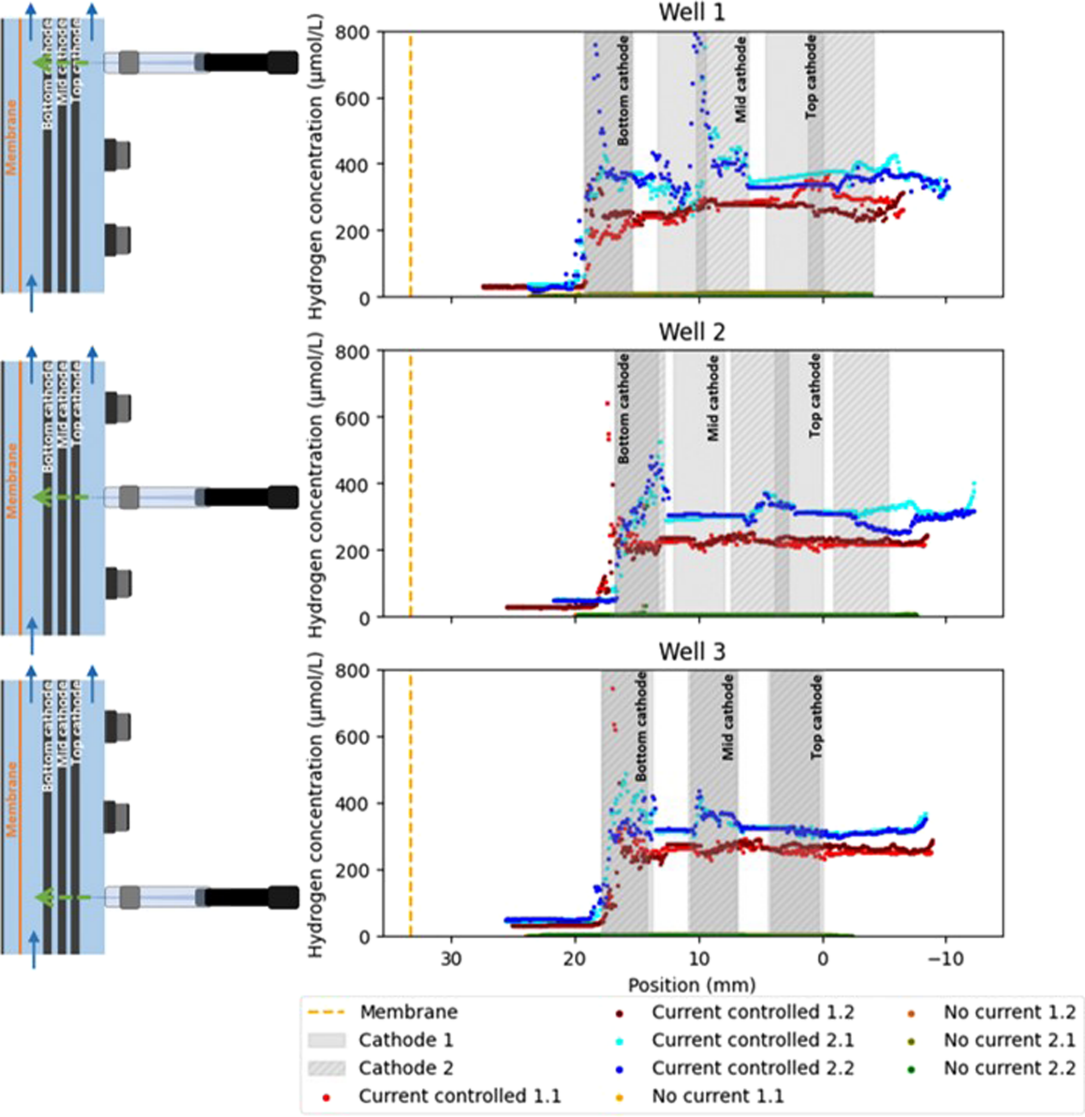
Profile of hydrogen concentration over the distance inside duplicate electrochemical reactors (1 and 2) operated at −200 mA and without current. On the left, schematics of the reactor is shown. The profiles were made twice in each reactor (.1 and .2).

With the new reactor design and profiling method, hydrogen concentration gradients were measured in the three wells of duplicate reactors with active catholyte recirculation (Figure 3). Prior to the profile, the sensors were calibrated according to the manual. Since the calibration was done at a lower temperature than the profile measurement, a temperature correction was performed for the conversion of the mA signal to the dissolved hydrogen concentration (SI section “protocol microsensor calibration”). After calibration, profiles of the hydrogen concentration distribution were made in duplicate with and without current to the reactor (Figure 3). To improve readability of the profile graphs, all figures show a schematic figure of the reactor orientation on the left side, corresponding with the visualization orientation in the figures. Without current applied to the electrochemical cell, no hydrogen was detected (Figure 3, OCV profiles). With applied current, the hydrogen concentration is low in the catholyte closest to the counter electrode (Figure 3, distance of 20–25 mm), and highest at the cathode closest to the counter electrode (Figure 3, bottom cathode). Two hydrogen measurements were done per reactor to investigate whether the piercing of the cathode layers in the first cycle would affect the local concentrations. Figure 3 shows that cycles 1 and 2 (.1 and .2) are similar, yet not exactly the same in all locations. Although the hydrogen was high in the bottom cathode, the theoretical maximum saturation concentration of hydrogen at the salinity and temperature used in this experiment (709 μmol/L) was not detected in duplicate. Differences between the duplicate measurements can be used to identify measurement interference by, e.g., gas formation.

To relate the local hydrogen concentrations to the distribution of local current at the different cathode layers, the applied current was measured over the resistances placed before each connection to the cathode layers. The current was not distributed evenly over the top, mid, and bottom cathode. A major part of the −200 mA supplied to the cathode was led to the bottom cathode closest to the counter electrode (±82%, Table S1).

### Correction for Electric Field Interference in Potentiometric Measurements

#### Measuring Electric Potential Offset vs Fixed External Reference Electrode

**Figure 4:**
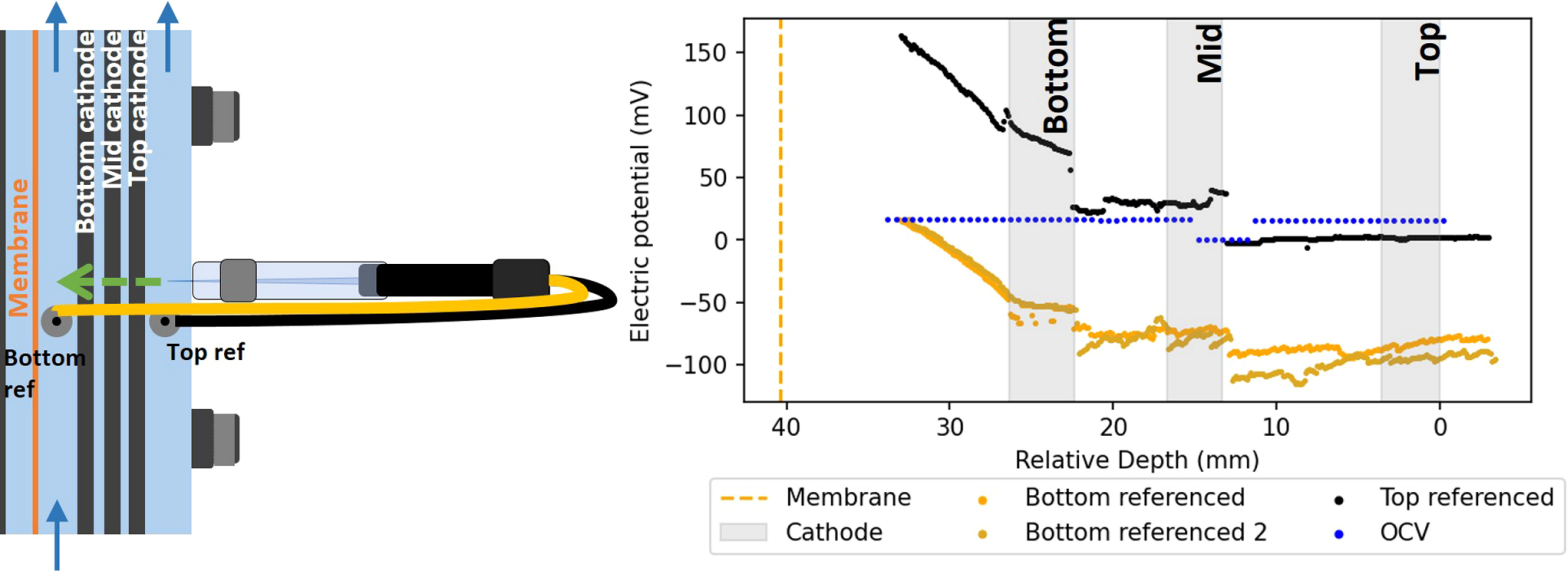
Profile of electric potential over the distance inside the middle well (left) of an electrochemical reactor operated at −50 mA (black, yellow, gold) and during open cell voltage control (blue). The electric field potential was measured as the potential difference versus the bottom reference electrode (yellow) and top reference electrode (black).

Since the current was not evenly distributed between the different cathode layers, it was expected that the local electric field would also show gradients over the different cathode layers. To investigate this, the EP (electric potential) sensor was used, which measures the potential difference between the microsensor tip and an external reference electrode; in this study, Ag/AgCl was used (Table 1). Since the EP sensor is also a Ag/AgCl electrode, the value of the electric field should be 0 when no electric field is present and increase with increasing electric field.^21^ To measure the electric potential in the electrochemical system, a profile was measured with the EP microsensor throughout the electrolyte and the cathode layers. First, a profile was measured without current applied to the system. During OCV, the electric potential difference between the sensor tip and the fixed reference electrode is constant at 20 mV with the exception of one jump to 0 mV around the middle cathode layer (Figure 4, blue dashed line). On the contrary, when the cathode is current controlled, the electric potential difference versus the same fixed reference electrode shows steep gradients and increases in jumps at each cathode layer when moving the sensor from the fixed reference (black line). Since most of the current was distributed to the bottom cathode, the local electric field was expected to be greater at the bottom electrode. There, a steeper gradient is seen throughout and below the bottom cathode layer (black line, left gray plane). The difference between the OCV and current controlled measurements shows that applying current affects the local electric field. With the fixed reference electrode positioned in this field of steep increase (bottom reference, yellow line), the gradient pattern is the same, with 0 mV offset when the moving electric potential electrode tip was (observed by eye) close to the bottom fixed reference electrode (depth 30 mm).

Although the difference between the black and yellow profile seems to be constant, the difference decreases from lower (33 mm, 150 mV) to higher locations (−4 mm, 100 mV) (Figure S6). The depth with offset 0 mV (30 mm) was determined to be right next to the bottom fixed reference electrode.

### ORP Profile Signal Corrected for Local Electric Field Potential

**Figure 5:**
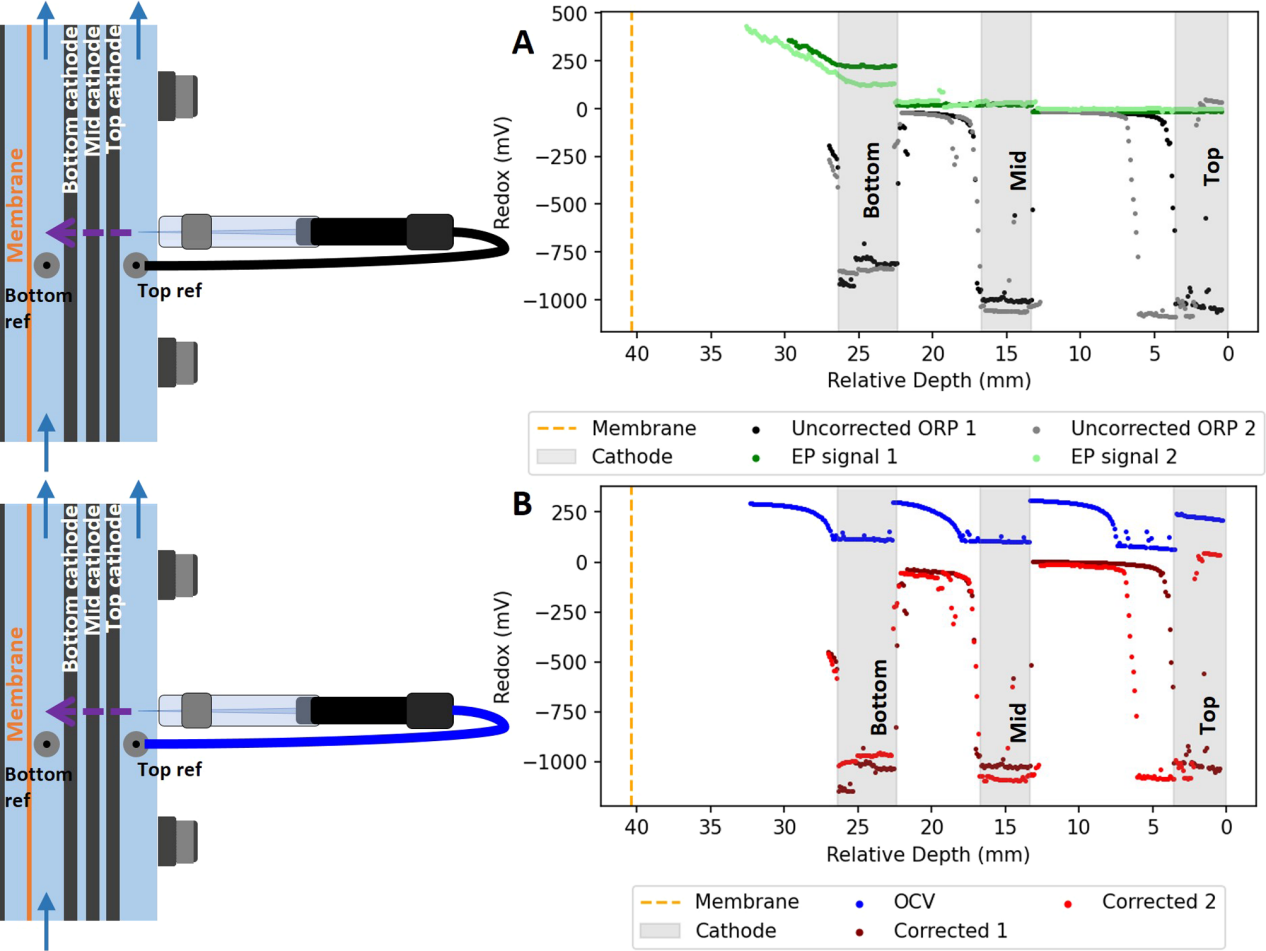
Duplicate profile of oxidation–reduction potential (ORP) over the distance inside an electrochemical reactor operated at −200 mA. The ORP was measured versus the top fixed reference electrode. The raw data from the ORP measurement measured versus the top reference electrode (A, black and gray) were corrected with the EP top referenced profile (A, green) to obtain a corrected ORP profile (B, red). The ORP profile was also measured with open cell voltage (B, blue). The duplicate measurements were performed in different reactors (1 and 2), and the cathode positions are only shown from one reactor (1) for viewing purposes.

Next to the EP sensor, the ORP microsensor also uses an external reference electrode (Table 1). Based on the EP profile (Figure 4), local mV offset signals can be expected in microsensor measurements with current applied to the system. Therefore, using the raw output data from potentiometric sensors would result in unreliable values. Damgaard et al.^21^ suggested that a local EP correction could be used to convert the potentiometric microsensor signals to accurate data. The ORP and pH microsensor used in this study were shielded against electric field disturbances with similar caging technique as used for the EP microsensor.^21^ Therefore, the three microsensors were expected to be disturbed by the electric field in similar ways. In this study, the hypothesis from Damgaard et al.^21^ was tested. Figure 5 shows the raw ORP data (black/gray) and the local EP (green) used for the ORP data correction (red) measured in two reactors (1 and 2). The correction was done by subtraction of the local EP difference versus the fixed top reference (Figure 5A, green) from the raw ORP signal measured versus the same fixed reference (Figure 5A, black/gray). To validate the accuracy of the correction, the cathode layer potentials were compared with the raw data. Since the cathode layers were connected in parallel to the potentiostat, the cathode layer potentials should be equal. This is indeed shown for the data corrected for the local EP with deviations of max 200 mV (∼15%) (Figure 5B, ‘Corrected’), but not for the uncorrected signal (deviations up to 360 mV, ∼28%), showing that the EP correction results in reliable ORP values. The corrected values could be compared to ORP without current applied to the system (Figure 5B, blue). It should be noted that the OCV profile and the duplicate ORP/EP profiles were measured in a different (but similar) reactor, so the exact cathode positions differed (top cathode was placed more to the left). Without current applied to the reactor, the ORP of the cathode layers is constant at 150 mV, while the catholyte ORP signal shows gradients toward 230 mV (Figure 5B, blue). All ORP values without current are less negative than with current.

### pH Microsensor Signal Interfered by Applied Current

**Figure 6:**
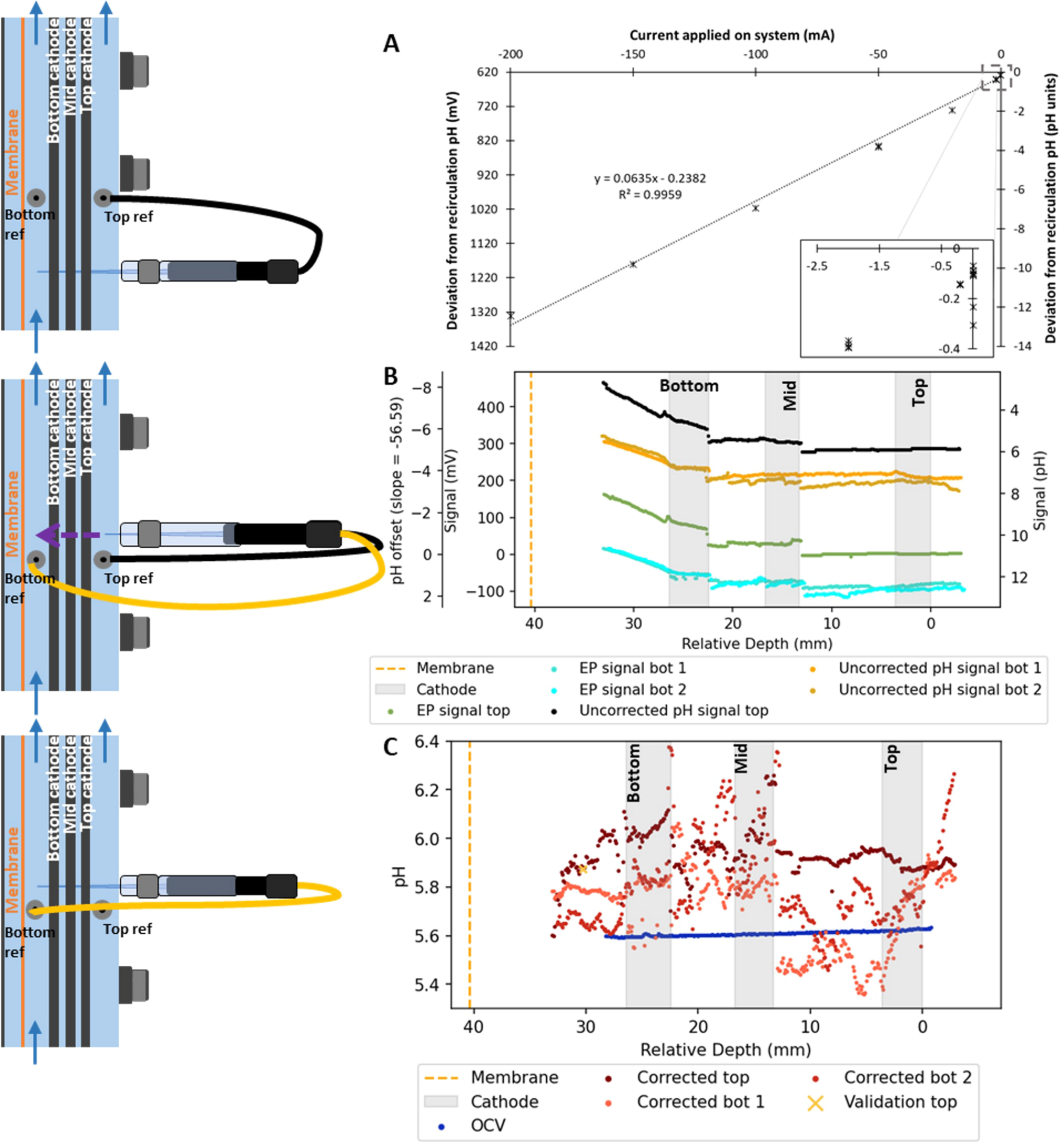
pH microsensor measurements. (A) Deviation between bulk pH and pH reported by the pH microsensor placed at the bulk inlet point of the cathode chamber at different cathode current magnitudes. The pH signal was measured versus the top fixed reference. (B, C) Profile of pH over the distance inside an electrochemical reactor operated at −50 mA. The pH was measured versus the top fixed reference electrode and twice versus the bottom fixed reference electrode. The raw data from the pH measurements measured versus the top (B, black) and bottom (B, yellow) reference electrode were corrected with the EP top (B, green) and bottom (B, light blue) referenced profile, respectively, to obtain corrected pH profiles (C, red). The pH profile was also measured with open cell (C, blue). Right after making the pH top referenced profile, the pH microsensor tip was placed next to the bottom reference electrode and logged over 7 min (C, yellow cross).

The pH microsensor also uses an external reference electrode (Table 1), so its mV signal was also expected to be interfered by the presence of an electric field. However, unlike the ORP measurements, the value around the cathodes could not be used as verification. Since the pH of the catholyte bulk recirculation is measured outside the electric field (Figure 1), this was used as a validation method in an experiment to investigate the magnitude of the signal deviation in relation to the current magnitude. Different current magnitudes were applied to the electrochemical system, while measuring the pH with a microsensor. To measure the deviation, the tip of the pH microsensor was placed at the influent port of the catholyte recirculation (Figure 6A, left). With this measurement, the microsensor pH could be compared to the recirculation pH. Figure 6A shows the deviation between the pH reported by the microsensor (with top reference at a depth of −5 mm, 35 mm from the pH microsensor tip) and the recirculation pH plotted against increasing cathode current.

When no current was applied to the system, a deviation between 0.06 and 0.3 pH units in signal was measured between the recirculation pH and the microsensor pH (Figure 6A). With applied current, the pH microsensor reported pH values lower than the recirculation pH. This offset increased with increasing current with a semilinear trend. At current values of −100 mA or more negative, the pH microsensor even reported negative pH values. Therefore, the signal deviates strongly from the recirculation pH with applied current.

### pH Microsensor Signal Corrected with Local Electric Potential

After determining the pH microsensor disturbance by the electric field at one point, a pH gradient profile was made with the fixed top reference (depth of −5 mm) (Figure 6B, black) and in duplicate with the fixed bottom reference (depth 30 mm) (Figure 6B, yellow). The signals showed a pattern similar to the signal from the electric field potential when measured with the same reference electrode point at −5 mm (top reference) (Figure 6B, green) or at 30 mm (bottom reference) (Figure 6B, light blue).

The local electric field correction was also applied to the pH microsensor measurements with the EP profiles measured from with the same fixed reference electrode as the pH profile (Figure 6C, red). To verify the local electric field potential correction, the pH was measured versus the bottom reference with the microsensor tip placed next to the bottom reference electrode (depth 30 mm), to ensure 0 mV offset (Figure 4) and logged over 7 min right after the pH measurement with the top reference (Figure 6B, black) (Figure S7). The average pH value during that period is indicated with a yellow cross (Figure 6C). The verification point lies exactly on the line of corrected pH values, showing the accurateness of the correction. The corrected values are more constant over the depth of the reactor and show a small gradient underneath the bottom cathode (depth 27 to 33 mm). The pH value in the bulk solution underneath the cathode (depth 33 mm) is similar to the bulk pH (5.7–5.9), while the pH is higher (up to ∼6.2) in the lowest two cathode layers (depth 13 to depth 30 mm). More away from the counter electrode, above 13 mm, the pH is again similar to the bulk pH value. Without applied current, the pH showed less gradients than with current (Figure 6C, blue and red).

### Intermittent Current or Distance to Reference to Allow Potentiometric Measurements

Next to the EP correction method, two additional methods to make profiles with potentiometric sensors are applying intermittent current or minimizing the distance to the reference electrode. When no current was applied between the electrodes, both the local EP and the pH offset were minimal (Figures 4 and 6A). Based on this insight, intermittent current was investigated as the method to measure with potentiometric microsensors. In theory, the values measured right after switching off the current should represent the actual value during applied current. To test this, the microsensor pH values were logged during intermittent current with the tip 35 mm from the external reference electrode. As validation, the microsensor pH values were also logged at the same location but with the external reference next to the tip (with 10 mm distance parallel to the electrode surfaces), with local EP of 0 mV. It was found that it took some time (at least 1 s) after stopping the current before the signal reached validated values representative for the situation with applied current. Simultaneously after stopping the current, the system gradients caused by the applied current disappeared and bulk conditions were measured. To obtain reliable values with potentiometric measurements during the intervals without current, the sensor should measure values that represent the situation with current on the system and not values that represent the bulk conditions, which are reached without applied current. For the systems described in this study, the intermittent current method was not reliable in some of the tests (SI section “pH microsensor measurement during intermittent current”). To determine the applicability of the intermittent current to measure with potentiometric microsensors, it is recommended to use the validation method described in SI section “pH microsensor measurement during intermittent current”.

## Discussion

### Microprofiling in Electrochemical Systems for Local Gradient Measurement

With the adapted setup, microsensor profiles can be made in the electrolyte and through the different porous electrode layers of the cathode chamber while keeping leak-free electrolyte recirculation. With the hydrogen sensor, local hydrogen concentrations could be mapped precisely (Figure 3). For measurements with potentiometric microsensors, an external reference electrode is used. Both the distance to the external reference (Figure 4) and the magnitude of the current (Figure 6A) influence the signal disturbance from microsensors with external reference electrodes.^8^ Potentiometric microsensor measurements in fields with low electric potential yet significant distance between the microsensor tip and the external reference electrode, as performed in earlier studies,^29,30^ could give seemingly plausible values, even though the electric field still causes an offset (Figure 6A). Since 59 mV corresponds with 1 pH unit, a 6 mV offset could already cause a measuring error of 0.1 pH unit. Therefore, the signal from microsensors with external reference electrodes needs to be corrected for this disturbance. The suggested correction with the local EP signal^21^ was tested and validated in this study. The corrected pH profiles show noise, but the corrected ORP profiles do not show this noise, indicating that the noise is not caused by the correction itself. Since the ORP profile shows equal potential values for all parallel connected cathode layers and the pH validation measurement at the location with EP of 0 mV showed the same value as the corrected profile, the EP correction for potentiometric microsensor measurements is reliable. Further proof of reliability can be gained from the duplicate measurements. The similarity between duplicate profiles show the reliability of the method, with small deviations that can be ascribed to either differences in conditions over the measuring time (>2 h) or to invasiveness of cathode piercing by the microsensors.

Another method to measure potentiometric signals in systems with high electrolyte resistance is minimizing the distance to the reference electrode.^8,22,31^ The mV offset between the electric potential microsensor and the fixed reference was 0 mV when the distance was minimized (with both tips at equal distance from the anode, with 10 mm distance between the tips parallel to the electrode surfaces), both with the top reference electrode (depth −5 mm) and the bottom reference electrode (depth 30 mm) (Figure 4). The bottom reference electrode was located in an area with a steep gradient of electric potential but still showed 0 mV offset at the minimum distance between the reference and the microsensor tip. In future studies, placing fixed reference electrodes at additional intersection points is recommended for validation purposes (see also SI section “Considerations for practical applications”). The potential ideal solution for potentiometric microsensor measurements would be the development of a combined sensor with an integrated internal reference electrode with a long thin tip that allows piercing soft materials. This sensor could be used for measurement with the least invasiveness in electrochemical systems. Unfortunately, this sensor is not yet commercially available.

### Microprofiling Shows Steep Local Gradients

With the method from this study, many useful insights were already gained. One insight gained here is that H_2_ is stripped likely due to the CO_2_ supply in the electrochemical system. The hydrogen concentration is low at the places close to the membrane and influent port (Figure 3). This indicates that a great part of the formed hydrogen is flushed out in the recirculation bottle of the system, where CO_2_ and N_2_ are continuously sparged to the reactor (Figure 1). Microsensor measurements can be used to test liquid mixing capabilities in optimized reactor designs by measurements of local substrate availability. Furthermore, the microsensor measurements showed that the local hydrogen concentration is the highest at the bottom cathode, corresponding with a great share (82%) of applied current towards that cathode layer. The catholyte recirculation distributes the formed hydrogen evenly through the cathode compartment, and the concentrations are still around 375 μmol/L inside and around the upper two cathode layers (depth of −5 to 25 mm). This hydrogen concentration is 1000 times above the required threshold reported in the literature for several hydrogenophilic bacteria^32^ (assuming a Henry coefficient of 7.7 × 10^–06^ mol/(m^3^Pa)^33^). Apart from the hydrogen profile, the ORP and pH profiles also gave interesting insights. The ORP profiles showed great differences with and without current, not only in the cathode but also inside the catholyte (Figure 5B). This indicates that applying a current to the system does not only change the reaction conditions within the porous cathode but also in the liquid around. The pH profile showed no gradient when no current was applied to the system, but it showed local differences when the current was applied to the system (Figure 6C). The local pH inside and around the cathode layers was higher than the bulk pH, presumably due to proton consumption for the hydrogen evolution reaction. A pH shift can change favorability for microorganisms. For example, the 0.3 pH unit increase causes a 5% decrease of the undissociated fatty acid fraction, this fraction can inhibit methanogenic activity.^34^ The insights of these microsensor measurements can be used to adjust the reactor conditions in such a way that allows them to be more favorable for desired microorganisms. For system optimization, fluid dynamic studies within microbial electrosynthesis systems are one of the key points.^35^ The results of this study indicated that hydrogen distribution in the system requires optimization. Microsensor measurements of local conditions are a helpful tool to study different electrode and flow designs and their effect on potential limiting conditions.

### Outlook for Microsensor Application Possibilities

The current distribution was mainly (82%) toward the bottom cathode, while hydrogen is available in all three cathode layers. Thus, different niches can be found within the cathode compartment and even within cathode layers with different local hydrogen concentrations at different depths. Between and within the cathode layers, different availability of substrates, electron donors, and products can be expected based on the profiling results. Several modeling studies have calculated the presence of limiting gradients of, e.g., pH^6^ and H_2_^36^ in biofilms. Thus, the development of a biofilm on the graphite fibers will affect the local conditions even more than in the abiotic situation shown in this study. Verification experiments with microsensors can serve as validation by determination of gradients of substrates, products, and local conditions within biofilms. Linking the different local conditions to the performance at the different spots can give many insights for optimization. For example, linking the local H_2_ and current to microbial activity is insightful for determining the dependence of the microbes on electrical current versus hydrogen as the electron donor. With the method presented in this paper, gradients of H_2_, O_2_, H_2_S, CO_2_, H_2_O_2_, NO_2_^–^, pH, ORP, and electric potential^19,21,37−45^ can likely be measured in stable electrochemical systems with or without biofilms under anaerobic or even aerobic conditions.

## Conclusions

This study showed the successful application of microsensors for measurement of gradients in electrochemical systems. The reactor with measuring wells placed perpendicular to the profiling direction allowed for profiling with electrolyte leak-free recirculating conditions. The presented manuals and video instructions will aid future users to apply this method. Profiles were made of local H_2_, electric potential, pH, and ORP in the electrolyte and for the first time throughout the porous electrodes. For the potentiometric microsensors, a local electric field potential correction is validated as a reliable method to correct for signal disturbance from the electric field. The use of these sensors can be extended to study biofilm gradients and local reactor conditions in electrochemical systems.

## Supporting Information Available

Additional figures with signal stabilization measurements, validation measurements with the potentiometric sensor, pH microsens or measurement during intermittent current, considerations for practical applications, and manuals for sensor calibration and profiling (PDF)
Movie file for constructing the graphite felt layers (MP4)
Movie file for making a microprofile (MP4)
Movie file for constructing the electrochemical reactor (MP4)
