# Supplementary material for: Methodology for In Situ Microsensor Profiling of Hydrogen, pH, Oxidation–Reduction Potential, and Electric Potential throughout Three-Dimensional Porous Cathodes of (Bio)Electrochemical Systems
Source: Anal Chem. 2023 Jan 30;95(5):2680–9. doi: 10.1021/acs.analchem.2c03121 (PMC9909735; doi:10.1021/acs.analchem.2c03121)
Supplement: Supplementary file 1 — ac2c03121_si_001.pdf [file ac2c03121_si_001.pdf]

Supporting information

Methodology for in-situ microsensor profiling of hydrogen, pH, ORP and electric potential throughout 3D porous cathodes of (bio)electrochemical systems

Sanne M. de Smit[1,2], Jelle J.H. Langedijk[1], Lennert C.A. van Haalen[1†], Shih Hsuan Lin[1], Johannes H. Bitter[2]* & David P.B.T.B. Strik[1]**

[1] Environmental Technology, Wageningen University and Research, Wageningen, the Netherlands

[2] Biobased Chemistry and Technology, Wageningen University and Research, Wageningen, the Netherlands

* email: harry.bitter@wur.nl

** email: david.strik@wur.nl

Figure	Description	Page
Figure S1	Detailed reactor configuration and measurements	S-2
Figure S2	Macroscope images of microsensor tips	S-3
Figure S3-S5	Microsensor response of hydrogen (S3), ORP (S4) and pH (S5) over time at different depths	S-4 to S-6
Figure S6	Difference between top and bottom referenced EP signal	S-7
Figure S7	pH microsensor measurement next to bottom reference	S-8

Section	Description	Page
Reactor operation	Reactor operation parameter settings	S-2
pH microsensor measurement during intermittent current	Validation of pH microsensor measurement with intermittent current method (incl Figure S8-S9)	S-8 to S-9
Considerations for practical applications	Important notes for practical applications	S-10
Protocol microsensor calibration	Protocol for the calibration of microsensors, with temperature and salinity correction of H_2 sensors (incl Figure S10)	S-10 to S-11
Protocol profiling	Step-by-step protocol for making a microsensor profile	S-12

Table	Description	Page
Table S1	Current distribution over cathode layers	S-3

Video	Description
movie_reactorconstruct	Video instruction on how to construct an electrochemical cell to allow *in situ* microsensor profiling
movie_cathodeconstruction	Video instruction on how to construct the cathodes
movie_profiling	Video instruction on how to insert and remove microsensor into or out of the reactor

Reactor operation

The reactor anolyte and catholyte were recirculated at 10 L/h. The total volumes of the catholyte and anolyte were respectively 360 and 310 ml. To provide similar conditions to biotic operation, the catholyte recirculation bottle was sparged with CO_2 and N_2 with rates of respectively 100 LN/d and 233.3 LN/d. The anolyte recirculation bottle was sparged with N_2 to remove oxygen produced at the anode. After the catholyte recirculation bottle, pH measurement (QMP108X, Q-is, Oosterhout, the Netherlands) and control at 5.8 (Ontwikkelwerkplaats, Elektronica ATV, the Netherlands) were placed in the recirculation ("recirculation pH"). The reactor was operated in a temperature controlled cabinet (30°C) in abiotic mode, microbial growth was prevented by leaving out ammonium from the medium. The catholyte had the same composition as the abiotic catholyte from de Smit, et al. [1], the anolyte consisted of 7.5 g/L $Na_2HPO_4.2H_2O$, 3.0 g/L KH_2PO_4, 0.05 g/L $MgSO_4.7H_2O$, 0.01 g/L $Ca(OH)_2$. The catholyte part of the reactor was operated in batch mode and the anolyte was fed to the reactor with a hydraulic retention time of 4 days. During Open Cell Voltage operation (OCV), the cathode and anode were physically disconnected by removing the connection cable. During continuous operation, the reactor was current controlled (-200 mA, -10 kA/m^3, unless stated otherwise) by a n-stat Ivium potentiostat (IVIUM, The Netherlands). The cathode potential was measured against a Ag/AgCl 3M KCl reference electrode (QM710X, Q-is, Oosterhout, the Netherlands), connected via a capillary, filled with gelified 3M KCl, positioned 5 mm above the top cathode (Figure S1A, cathode reference). After startup, the reactor cathode potential was left to stabilize for at least 24 hours to perform measurements in steady state conditions.

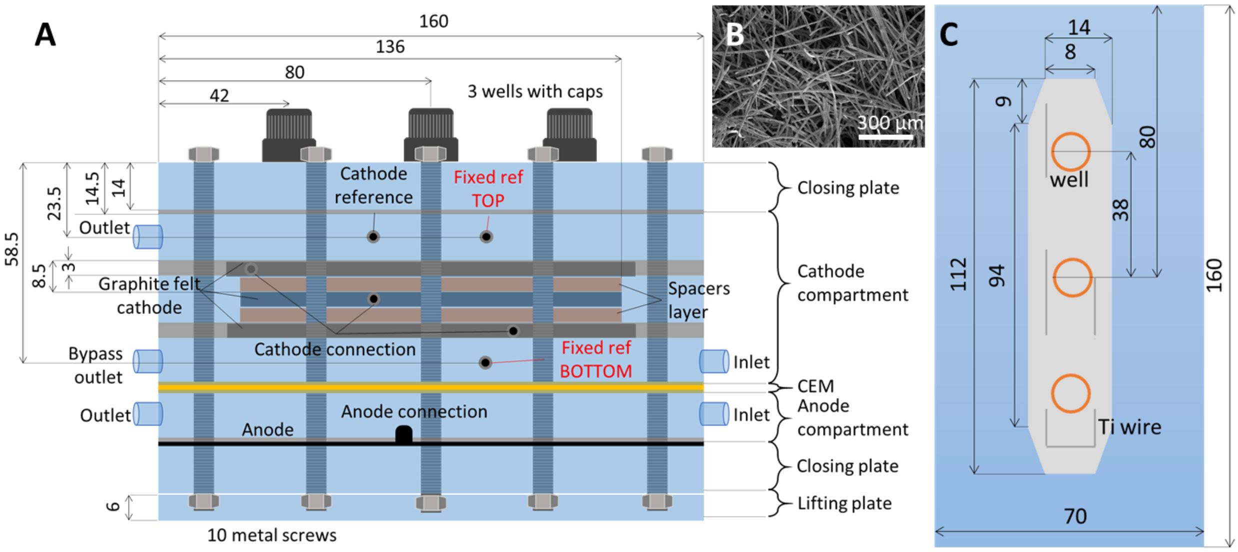

Figure S1. Electrochemical reactor with three cathode layers and three profiling wells (A). The graphite felt used as cathode had a 3D structure (B, SEM image). (C) A top view of the cathode flow through plate shown with the location of the three measuring wells. Measurements are shown in mm.

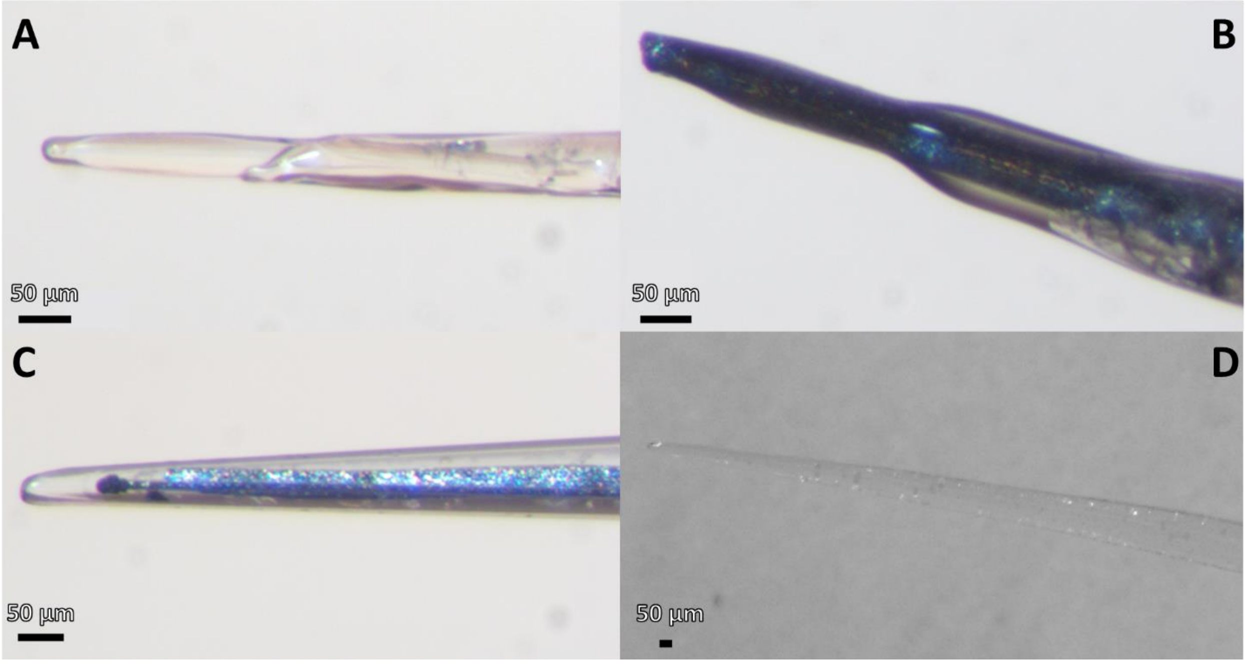

Figure S2. *Macroscope images of the tips of the pH (A), ORP (B), hydrogen (C) and electric field potential (D) microsensor.*

Table S1. *Current distribution over three graphite cathode layers connected in parallel at different distances from the anode (Figure 1A).*

	Cathode layer	*Current (mA)*	*Stdev during 3 days (mA)*
Reactor 1	*Top*	*-17.2*	*1.8*
	Middle	*-20.3*	*1.3*
	Bottom	*-163.8*	*0.3*
Reactor 2	*Top*	*-16.1*	*3.1*
	Middle	*-19.4*	*4.6*
	Bottom	*-164.1*	*6.5*

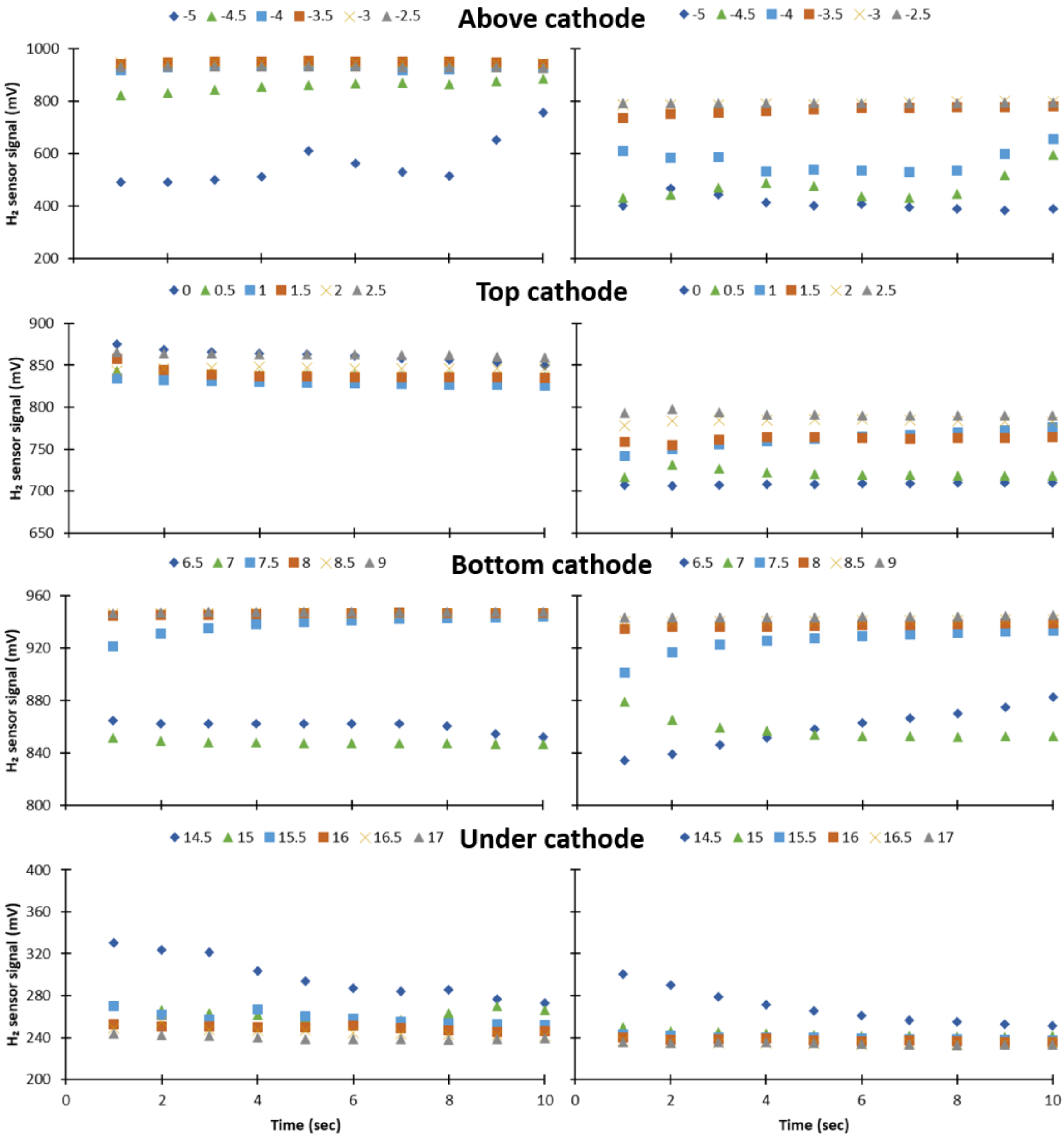

Figure S3. *Hydrogen microsensor response over 10 seconds measured in duplicate (left and right) at different depths, indicated in legend (normalized to cathode top). The response times were measured in an abiotic reactor with a 9 mm graphite felt cathode controlled at -0.85 V vs Ag/AgCl.*

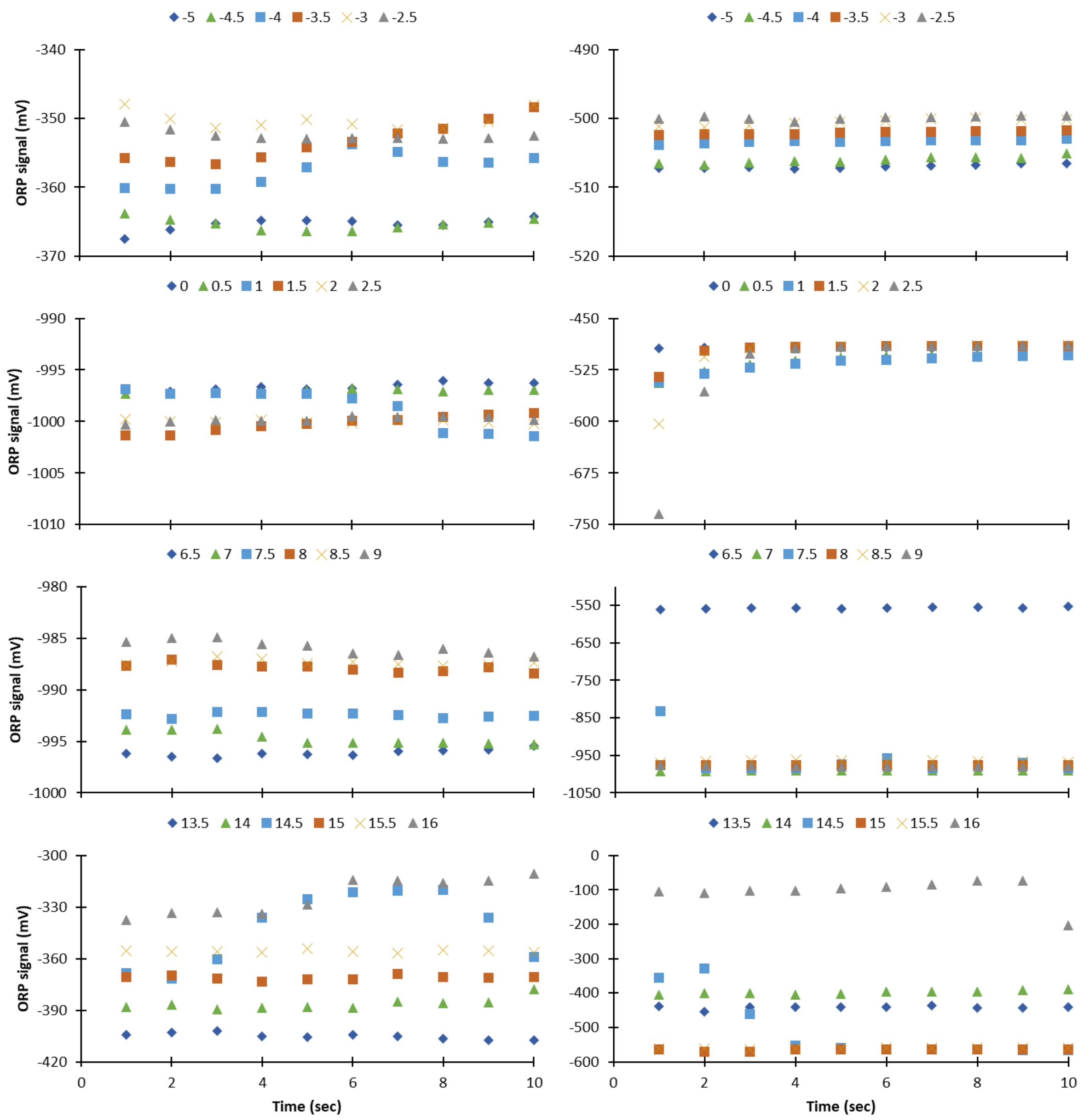

Figure S4. *ORP microsensor response over 10 seconds measured in duplicate (left and right) at different depths, indicated in legend (normalized to cathode top). The response times were measured in an abiotic reactor with a 9 mm graphite felt cathode controlled at -0.85 V vs Ag/AgCl.*

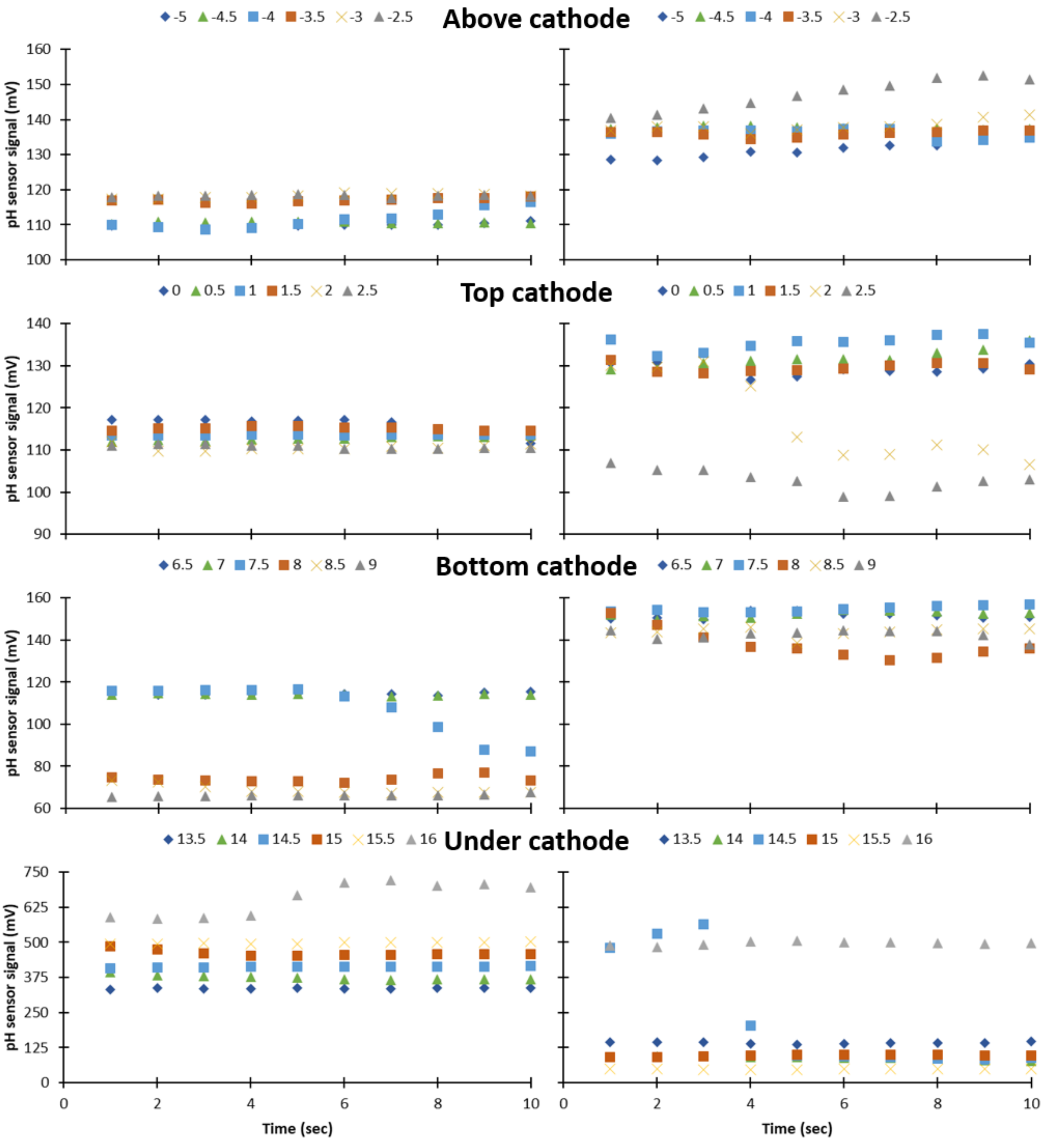

Figure S5. *pH microsensor response over 10 seconds measured in duplicate (left and right) at different depths, indicated in legend (normalized to cathode top). The response times were measured in an abiotic reactor with a 9 mm graphite felt cathode controlled at -0.85 V vs Ag/AgCl.*

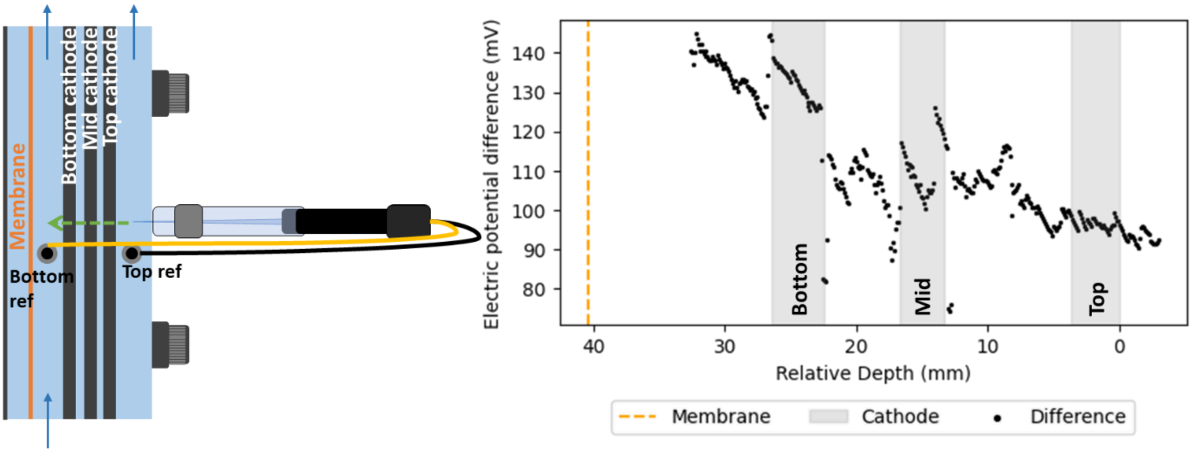

Figure S6. *Difference between electric potential measured versus the fixed top reference (left, black) and fixed bottom reference (left, yellow) over the depth of the reactor.*

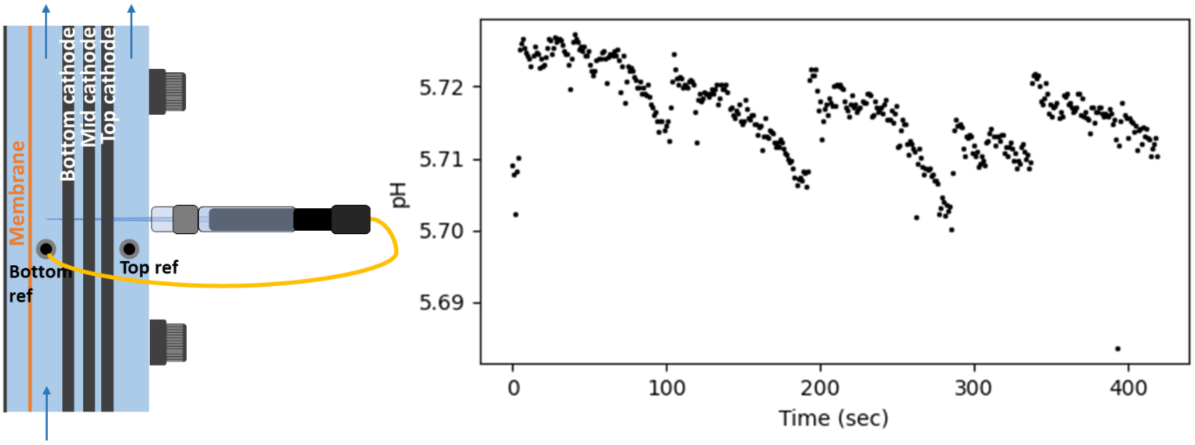

Figure S7. *pH microsensor response over 7 minutes with tip placed right next to the bottom reference electrode (depth 30 mm). An abiotic CO_2-fed reactor was controlled at -50 mA during this experiment.*

pH microsensor measurement during intermittent current

The offset between the pH microsensor and the recirculation pH increased with increasing current (Figure 8). Based on this result, it was hypothesized that shortly disconnecting the current supply would remove the interference and result in reliable values from the microsensor. To test this, intermittent current was applied to the cathode, whilst measuring the pH. Current was applied intermittent to the cathode, with 40 s of control at -200 mA alternated by 7 s of open cell voltage. The pH microsensor was placed in the reactor with the tip right next to the top fixed reference electrode. The signal of the microsensor was measured both versus the top fixed reference and versus the bottom fixed reference at 35 mm from the microsensor tip (Figure S7, left).

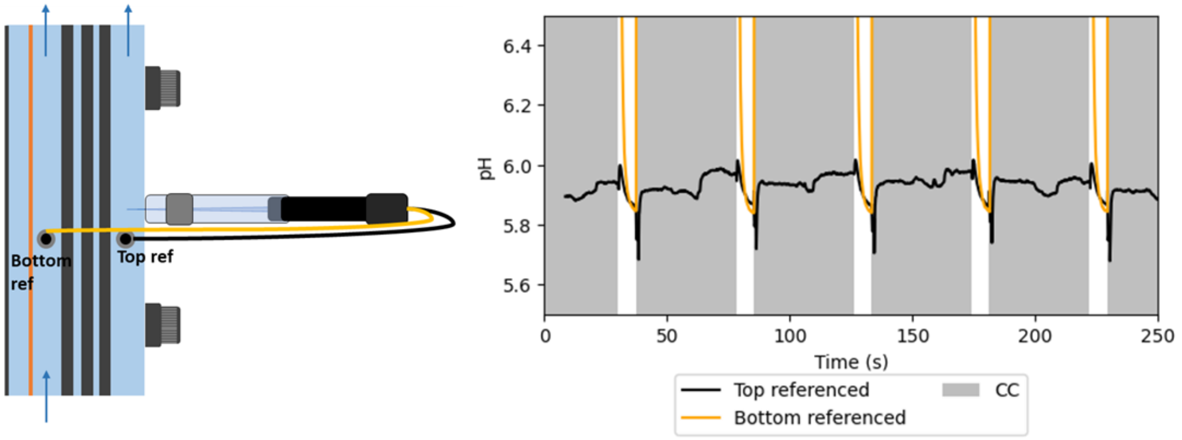

Figure S8. *pH microsensor measurements during intermittent current (right). The cathode current was -200 mA for 40 s (grey planes) and turned off for 7 sec (Open Cell Voltage, white planes). The pH microsensor tip was placed next to the top fixed reference electrode positioned at -5 mm (left). The signal with the top reference (black) is overlayed with the signal with the bottom reference electrode positioned 35 mm lower than the top reference (yellow).*

When the pH microsensor tip was measured with the top fixed reference electrode, the signal during current control and during open cell voltage is similar (Figure S7, black line). This shows that that signal was unaffected by the applied current and thus reliable[2,3]. During the 7 sec open cell voltage, the signal dropped approximately 0.1 pH unit (Figure S7). When no current is applied to the cathode, no additional hydrogen is formed from the protons, while the continuous recirculation causes mixing with the bulk liquid. Therefore, after stopping the current supply, the local pH will eventually be equal to the bulk pH (5.8).

When the pH microsensor tip was measured against the bottom fixed reference electrode, the pH signal gave a value around 16 to 17 during current controlled operation (Figure S7, grey planes, yellow line). This corresponds to approximately 600 mV offset (59 mV per pH unit), which was also the difference measured between the two fixed reference electrodes (bottom and top, Figure S6 left). After the switch to open cell voltage operation, the pH microsensor first shows a rapid decrease and then a slower decrease (white planes, yellow line).

For the measurement shown in Figure S6, the potentiometric sensor measurements with different reference electrode positions reach the same value during open cell voltage. This supports the use of intermittent current as a method to measure reliable pH during the intervals without supplied current, representative for the current controlled situation. However, in more mature systems or systems with a grown biofilm, the potentiometric sensor measurements with different electrode positions did not reach the same value during open cell voltage. This suggests that 7 seconds was found too short for the electric interference to disappear and the bottom referenced signal to reach reliable values (Figure S8). Applying open cell voltage for longer time is likely to make the electric interference disappear. However, applying longer open cell voltage time would result in the gradients to disappear and the bulk pH would be measured. In that case, the measurement would not be representative anymore for the situation with applied current.

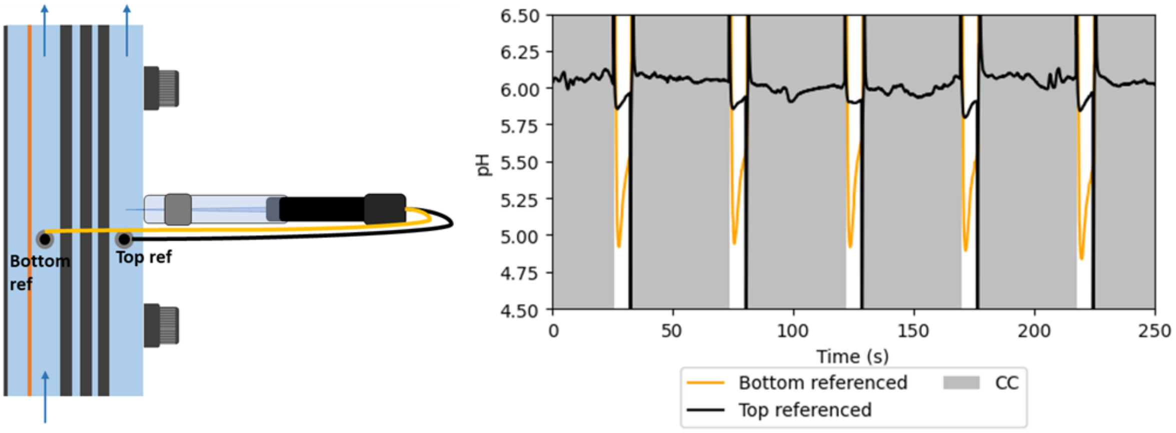

Figure S9. *pH microsensor measurements during intermittent current (right). The cathode current was -200 mA for 40 s (grey planes) and turned off for 7 sec (Open Cell Voltage, white planes). The pH microsensor tip was placed next to the top fixed reference electrode positioned at -5 mm (left). The signal with the top reference (black) is overlayed with the signal with the bottom reference electrode positioned 35 mm lower than the top reference (yellow).*

For different systems, a similar experiment as described here can be performed to determine whether intermittent current can be applied to perform potentiometric measurements. Additionally, the response of the electric potential microsensor after switching off the current can be measured to determine the time before electric interference disappears. When this time is short enough to still measure local conditions representative for the situation with applied current, the intermittent current method is applicable. An additional requirement is that the pH microsensor signal response time is also fast enough to measure representative values for the situation with applied current. Besides the reliability of the potentiometric measurement, the effect of applying intermittent current on (microbial) processes within electrosynthesis systems should be considered before using the method[4-6].

Considerations for practical applications

When using the local electric potential correction, placing fixed reference electrodes near positions of interest is recommended to allow verification measurements and ensure high accuracy at the points of interest. Additionally, the location of the potentiometric microsensor tips needs to be precise. Figure 6 shows that especially at locations with high current (depth 22-33 mm), the electric potential can differ substantially per location. Thus, a deviation in the determination of the exact microsensor tip location will cause a deviation in the correction. Measuring the relative distance between the tips of two sensors (e.g. EP and potentiometric sensor of interest) can be accurately performed under a macroscope. Another important factor is system stability. Since the local electric field potential measurement involves profiling the reactor with two different sensors (sensor of interest and electric potential sensor), the local electric field needs to be stable between these two profiling cycles. Interference by e.g. gas bubbles should be avoided.

Protocol microsensor calibration

Note: all calibrations can be performed with the microsensors still in the plastic protective cover. Perform calibration before (and after) each profiling run, to check similarity of calibration results

Hydrogen sensor

Calibration

- Connect sensor to amplifier
- Polarize the sensor at the value described in the manual (latest value 100 mV), wait until the signal is stable
- Start software; connect to motor and amplifier
- Place sensor in closed glass container with demi water (or measuring matrix, recommended), gas sparger connection and offgas connection for calibration *NB: ensure no gas could build up*
- Add calibration point 0
- Retract the sensor from the liquid
- Connect sparging connection to H_2 gas bottle and flush for approximately 15 min
- Insert the sensor back into the liquid
- Ensure the liquid within the protective sleeve is well mixed with the sparged and saturated bulk (e.g. by moving sensor up and down)
- When mV signal is stable for at least 5 min, add calibration point corresponding to saturation (µmol/L)

Correction hydrogen saturation point for salinity and temperature

The maximum saturation point for hydrogen needs to be corrected for temperature and salinity. After profiling, the highest calibration point needs to be replaced with:

1. mV corrected for the temperature of the measurement

The mV signal given when measuring a maximal saturated solution is dependent on temperature. This dependence is sensor dependent and thus needs to be remade for every new sensor. The dependence can be determined by measuring the mV of the signal in a maximal saturated solution at different temperatures (Figure S9).

2. Saturation concentration at the salinity (and temperature) of the measurement solution

The saturation concentration can be found in literature[7], once the salinity of the measured solution is known.

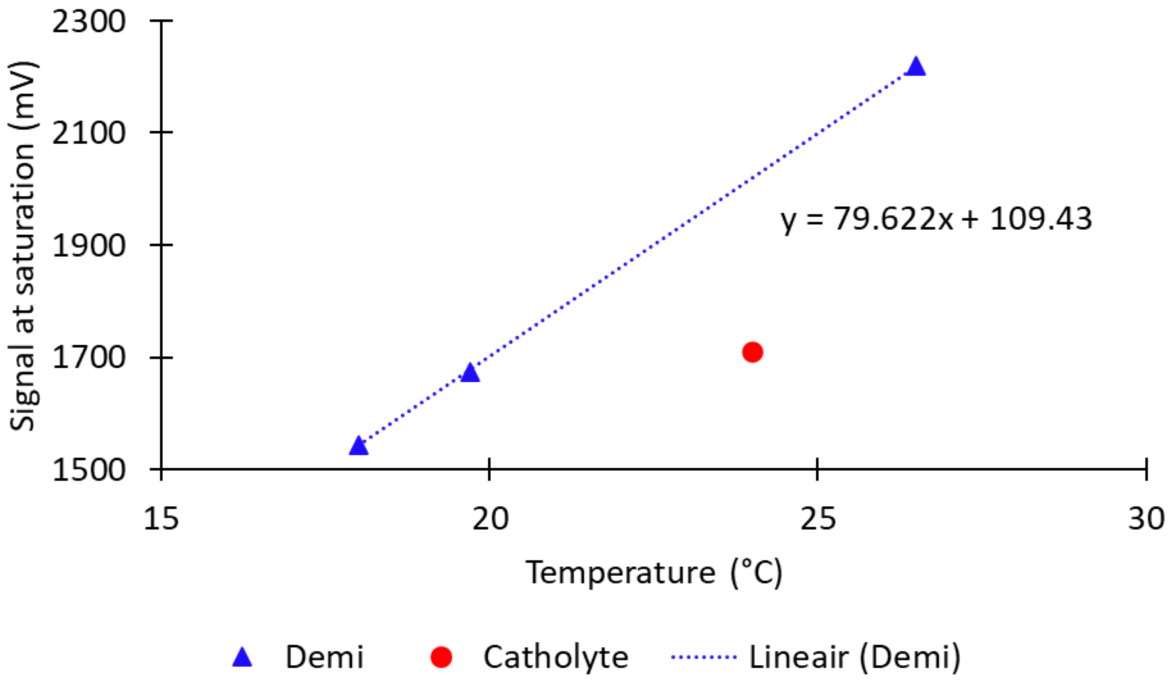

Figure S10. *Millivolt signal of H_2-signal at maximum saturation at three different temperatures in demi water and catholyte. This line can be used to correct for temperature when calibration temperature differs from measuring temperature.*

pH sensor

The pH measurements are temperature sensitive. Therefore, it is important to store the buffers at the temperature of your measuring conditions.

- Connect the pH microelectrode to the amplifier and connect a reference electrode to the microsensor cable
  in this study Ag/AgCl was used, make sure the reference is the same for measuring and calibrating; the reference was connected to a glass capillary with a porous membrane to fit the reference electrode together with the reference electrode in the buffer tubes
- Start software; connect to motor and amplifier
- Rinse pH microelectrode (carefully) and reference electrode tip (capillary)
  NB: don't wipe glass microelectrode tip with paper, only wipe plastic cover
- Place both the microelectrode and reference electrode tip (capillary) in the buffer tube
- Starting from 7, calibrate with multiple buffers in the range of interest (in this study: 7, 4, 6, 9, 10)

Sensor heights and motor tool

Note that the sensors have different lengths. Depending on the sensor length, the height of the sensor in your system will be different when positioned at the same height of the motor tool. Therefore, correct the motor tool heights according to the sensor lengths. It is recommended to always measure a new microsensor since the handmade lengths might differ.

Protocol profiling

1. Move the step motor (A) with motor control software to the highest position. This can be skipped if the step motor was return to position 0 after latest profiling
2. Lift the micromanipulator manually to highest with knob B (B)
3. Fix MES cell to the tilting ground plate with glue clamp to with the microsensor clamp (C) placed above the measuring well of interest
4. Widen Microsensor clamp (C)
5. Connect and perform corresponding calibration on selected microsensor, with protective tube on. (see appendix protocol microsensor calibration)
6. Place the microsensor on sliding rail plate (milled wooden shelf) and remove the plastic protection cover by sliding it through the groove while holding the microsensor at the head to prevent touching the wood with the glass tip
7. Adjust the profiling sleeve to the microsensor by sliding the microsensor over the groove into the sleeve (the sleeve should be stick to the plastic neck of the microsensor with residual silicon grease, apply some if not)
8. Set aside in a safe place for mounting

Mounting microsensor (to be done fast, usually within 5 min)

9. Stop both recirculation pumps (and if needed acid/base supply)
10. Close the cathode inlet and outlet valves of MES cell to detach from rest of the recirculation.
11. Open cap of target profiling well
12. Clamp the microsensor at its head, align the top of the microsensor head with top of the clamp (as precise as possible, this will determine the positioning of the cathode). Be careful not to touch sensor tip with anything
13. Move the planer position of center with knob D (D, two knobs) to target the profiling well, with sensor tip as reference
14. Drop the micromanipulator to lowest manually with knob B (B). Make sure microsensor enter the well without touching
15. Slide down to detach the profiling sleeve with plastic neck. Avoid touching the glass part of the microsensor. Screw the sleeve to the well handtight
16. If necessary: attach N_2 flushing tube to the flushing spot
17. Move microsensor to the vertical position with a small gap between plastic neck and sleeve tube (position depends on motor settings and microsensor-well combination)
18. Again, adjust the planer position of center with knob D to target the profiling well, but this time with plastic neck as reference. Apply extra silicon grease to the gap, before finishing the round, detach the N_2 flushing and replace with closing part
19. Move the microsensor deeper with software to complete the seal. Make sure the forwarded distance is long enough to provide resistance to avoid leakage. You can apply grease while microsensor is moving deeper to provide more seal
20. Make sure the whole periphery is sealed with silicon grease, no liquid or gas escape is formed

Testing the seal

21. Open the cathode inlet and outlet valves of MES cell to connect to rest of the recirculation
22. Start catholyte recirculation, observe for a minute if catholyte is leaking from the sleeve
    - Leaking -> Stop recirculation, close valve, and repeat from step 19
    - Not leaking -> go forward
23. Move Microsensor to the micro-profiling safe position and ready for micro-profiling.

General reminder for all microsensors

- Loosen the clamp and turn microsensor 90° to target new piercing site between each micro-profiling cycle. Do this in safe position and mind not to change the vertical position of microsensor. Apply new silicone grease before starting next cycle.

- Parameters can be changed during profiling operation, e.g. Figure 5. To do so, pause and change the parameters (end, wait, measure, replicate), then resume. The profiling operation will restart at the latest position. Note that the new parameters will only be applied for in measurements coming onward.

References

(1) de Smit, S. M.; Buisman, C. J.; Bitter, J. H.; Strik, D. P. *ChemElectroChem* **2021**, *8*, 3384-3396.
(2) Damgaard, L. R.; Risgaard-Petersen, N.; Nielsen, L. P. *J. Geophys. Res.: Biogeosci.* **2014**, *119*, 1906-1917.
(3) Beyenal, H.; Babauta, J. *Productive Biofilms* **2013**, 235-256.
(4) Lavender, M. B.; Pang, S.; Liu, D.; Jourdin, L.; Ter Heijne, A. *Bioresource Technology* **2022**, *347*, 126650.
(5) Pereira, J.; Mediayati, Y.; van Veelen, H. P. J.; Temmink, H.; Sleutels, T.; Hamelers, B.; Ter Heijne, A. *Biofilm* **2022**, *4*, 100064.
(6) Caizán-Juanarena, L.; Sleutels, T.; Borsje, C.; ter Heijne, A. *Renewable Energy* **2020**, *157*, 782-792.
(7) Wiesenburg, D. A.; Guinasso Jr, N. L. *Journal of chemical and engineering data* **1979**, *24*, 356-360.
